# Climate and landscape features associated with successful *Wolbachia* replacement in the municipality of Rio de Janeiro, Brazil

**DOI:** 10.1186/s13071-026-07602-y

**Published:** 2026-08-04

**Authors:** Katie Tiley, Katie Milligan Susong, Steffen Knoblauch, Kathleen M. O’Reilly, Oliver J. Brady

**Affiliations:** 1 Centre for Mathematical Modelling of Infectious Diseases, London School of Hygiene & Tropical Medicine, London, UK; 2 Department of Infectious Disease Epidemiology and Dynamics, Faculty of Epidemiology and Population Health, London School of Hygiene & Tropical Medicine, London, UK; 3 Division of Biosciences, University College London, London, UK; 4 Interdisciplinary Centre for Scientific Computing, Heidelberg University, Heidelberg, Germany; 5 Heidelberg Institute for Geoinformation Technology, Heidelberg, Germany

**Keywords:** *Wolbachia* replacement, *Aedes aegypti*, Modelling, Spatial heterogeneity, Rio de Janeiro

## Abstract

**Background:**

*Wolbachia* replacement is a promising strategy for reducing arboviral transmission by *Aedes aegypti*. *Wolbachia* infection is refractory to arboviruses, including dengue and chikungunya, and *Wolbachia* spreads in mosquito populations by maternal transmission and cytoplasmic incompatibility, whereby mating between infected males and uninfected females produce nonviable offspring. Deployment programmes seek to establish stable, high *Wolbachia* prevalence through the release of *Wolbachia*-infected mosquitoes; however, prevalence varies spatially, particularly in complex urban environments where fine-scale heterogeneity in temperature and built environment may influence mosquito population dynamics. Here, we investigate how climate and landscape features are associated with heterogeneous *Wolbachia* replacement outcomes using *w*Mel prevalence data from releases in the municipality of Rio de Janeiro, Brazil (2017–2019).

**Methods:**

Across a 26-month period, 500 × 500 m spatial units were clustered into four distinct replacement trajectories: unsuccessful, low, moderate, and high success, defined using predicted *Wolbachia* prevalence time series generated from cell-level binomial regression models and grouped using k-means clustering. Associations were explored between each trajectory and features related to *Ae. aegypti* habitat suitability, climate sensitivity, flight barriers, and *Wolbachia* deployment barriers using a multinomial generalised additive model (GAM). We used forward selection based on Akaike Information Criterion, explicit consideration of spatial autocorrelation and screening for collinearity among covariates. Of the 23 covariates examined, four covariates were retained: proportion of tall buildings (% > 6 m in height), average air temperature (°C), average elevation (m above sea level), and average daily rainfall (mm).

**Results:**

Results show high replacement success was mostly associated with areas with fewer tall buildings [*β* = −0.93 (95% CI −1.43, −0.44), *P* < 0.001] and higher temperatures [*β* = 1.11 (95% CI 0.59, 1.62), *P* < 0.001], while higher elevation and lower rainfall were associated with poorer outcomes. These associations may also reflect unmeasured socioeconomic or infrastructure variation, and context-dependent temperature effects on mosquito and *Wolbachia* fitness. Model-based predictions indicate non-linear dynamics, with areas of more tall buildings and higher temperatures showing divergent predicted outcomes towards either high success or failure rather than intermediate replacement. Residual spatial clustering suggests additional neighbourhood-scale processes not captured by the available covariates.

**Conclusions:**

Outcomes in high-rise and topographically complex areas appear less likely to stabilise at intermediate replacement. Such areas may require higher release densities, targeted supplemental interventions, and more intensive monitoring, although these remain as hypotheses requiring empirical evaluation. Through iterative modelling, it is possible to support rapid hypothesis identification to inform field tests and operational refinement as more evidence becomes available. These findings reinforce the role of urban permeability, vertical structure, and local environmental conditions in shaping the outcomes of *Wolbachia* replacement programmes.

**Graphical Abstract:**

**Figure:**
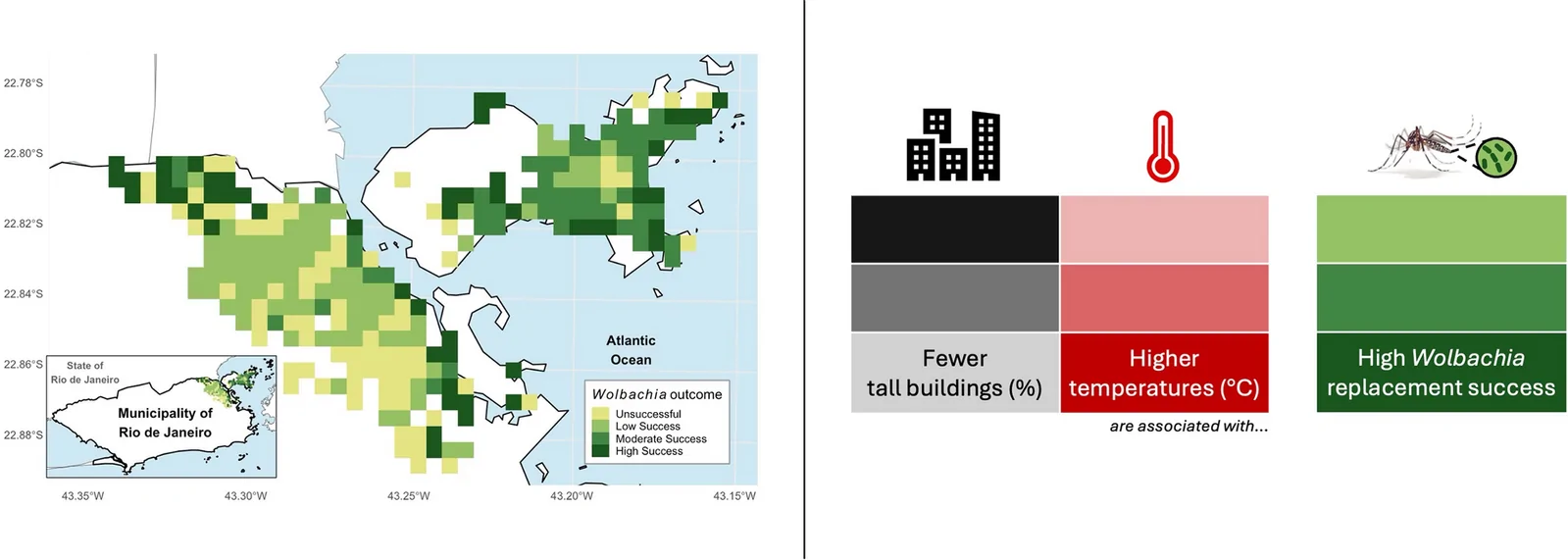

**Supplementary Information:**

The online version contains supplementary material available at https://doi.org/10.1186/s13071-026-07602-y.

## Background

*Aedes aegypti* are the primary vectors of dengue virus (DENV) across tropical and subtropical regions [1], where dengue imposes a substantial public health burden, with an estimated 96 million cases, 1.9 million DALYs and approximately 9,000 deaths annually [2]. The global distribution of *Ae. aegypti* is expanding [3], exacerbated by increased urbanisation [4] which increases availability of artificial breeding sites [5, 6 ].

While dengue vaccine development is promising [7–10], vector control remains a cornerstone of dengue control [2]. Conventional interventions include insecticides [11], larvicides [12], and limiting biting and reducing breeding habitats through community engagement and public works development [13, 14 ]. In recent decades, *Wolbachia* replacement has garnered interest as an intervention based on transinfection of *Ae. aegypti* with non-native *Wolbachia* strains (e.g. *w*Mel) which reduce dengue transmission in vector populations. *Wolbachia* are heritable intracellular bacteria which evolved as reproductive parasites in many arthropods [15], proliferating within populations through cytoplasmic incompatibility, whereby *Wolbachia*-infected males and uninfected females produce nonviable offspring [15–17]. Crucially, laboratory experiments demonstrate *Ae. aegypti* mosquitoes transinfected with *Wolbachia* exhibit refractoriness to dengue, Zika, and chikungunya viruses [18–22].

Experiments demonstrate that releasing *Wolbachia*-infected *Ae. aegypti* into uninfected populations results in successful replacement, i.e. population *Wolbachia* infection prevalence of > 90%, within a few generations [18, 23 ]. Theoretically, once *Wolbachia* infection prevalence crosses a critical population threshold, heritability and cytoplasmic incompatibility lead to continued proliferation without the need for additional intervention [17]. This threshold depends on strain characteristics, host fitness costs, maternal transmission efficiency, and local ecological conditions. Field releases have shown that *Wolbachia* replacement can achieve high, self-sustaining prevalence, but releases in some settings have also shown sustained intermediate prevalence (population infection prevalence < 60%), spatially heterogeneous (‘patchy’) replacement, or post-deployment loss of infection [20, 24, 26 –]. Imperfect *Wolbachia* replacement may still offer some benefits, intermediate replacement is reported to be associated with intermediate dengue transmission control in some settings [22]. Concerningly, deployments in the municipality of Rio de Janeiro found *Wolbachia* replacement is negatively correlated with dengue incidence [22], suggesting that areas with higher dengue transmission may present ecological or operational conditions less favourable to *Wolbachia* replacement. Understanding the factors associated with successful replacement could help identify opportunities to improve deployment effectiveness.

*Wolbachia* infection can impose context-dependent fitness costs on *Ae. aegypti*, including reduced egg viability, altered development, and reduced stress tolerance [27–35]. However, there are uncertainties on the influence of landscape features on *Wolbachia* infection dynamics. Spatial barriers which limit mosquito dispersal, such as highways [36, 37 ], and sudden changes in mosquito population densities [38] negatively impact *Wolbachia* spread. *Ae. aegypti* life history traits and breeding habitat availability are impacted by environmental factors such as temperature, humidity and rainfall [39, 40 ], vegetation cover [41, 42 ], and anthropological infrastructure [43–46]. Abrupt changes in climate, such as strong seasonal effects or extreme weather events, may also limit replacement [47–49]. For example, high temperatures from heatwaves may reduce bacterial density and lead to *Wolbachia* loss [50–52]. Researchers have speculated this is one reason for the unstable replacement reported after field deployments in Vietnam [25], however, the authors explicitly suggest undetermined elements of the built environment may affect *Wolbachia* replacement since replacement has been successful in other settings with analogous temperature profiles. In the case of Colombia [24, 53 ] and Brazil [20, 26 ], researchers suggest variable release intensity, due to logistical deployment limitations, and significant baseline variation in *Ae. aegypti* populations may have contributed to spatially heterogeneous replacement. Improving our understanding of landscape features on *Wolbachia* infection dynamics is essential for realising the maximum efficacy of *Wolbachia* replacement in diverse settings.

Mathematical models have been used to explore how release strategy, mosquito dispersal, habitat quality and fitness costs influence *Wolbachia* establishment across heterogeneous environments [48, 54, 58 –]. Using publicly available data of deployments in the municipality of Rio de Janeiro, we develop a multinomial generalised additive model (GAM) which explores factors associated with *Wolbachia* infection prevalence trajectories to answer the research question: to what degree do climate and landscape features shape spatially heterogeneous *Wolbachia* replacement?

## Methods

### Releases in the municipality of Rio de Janeiro

After an initial pilot in 2014, described in Garcia et al. [59], 67 million *Wolbachia*-infected mosquitoes were released across an 86·8 km^2^ area in the municipality of Rio de Janeiro in five stages (across three release zones) between August 29, 2017, and December 27, 2019. The pilot identified widespread insecticide resistance in baseline *Ae. aegypti* populations; thus, released mosquitoes were locally backcrossed with an insecticide-resistant *w*Mel strain. A network of BG-Sentinel traps (*n* = 1168) was used to monitor *w*Mel prevalence in *Ae. aegypti* every 2 weeks over the release period.

This study uses public data provided by Ribeiro dos Santos et al. [22], available at https://github.com/pdgcam/wMel_Rio_dengue_chik, accessed March 5, 2025. The data include the number of traps/collections, *Ae. aegypti* captured, mosquitoes tested positive for *w*Mel, and proportion *w*Mel. The data are spatially aggregated to a grid of raster cells (*n* = 396), each 500 × 500 m, with an irregular boundary and temporally aggregated monthly from August 2017 until September 2019 inclusive. Although releases continued until December 2017, the publicly available dataset covers the period up to September 2019 and therefore all analyses are restricted to this timeframe. See data source [22] for complete details of data collection.

### Outcome: wMel replacement trajectories

**Fig. 1:**
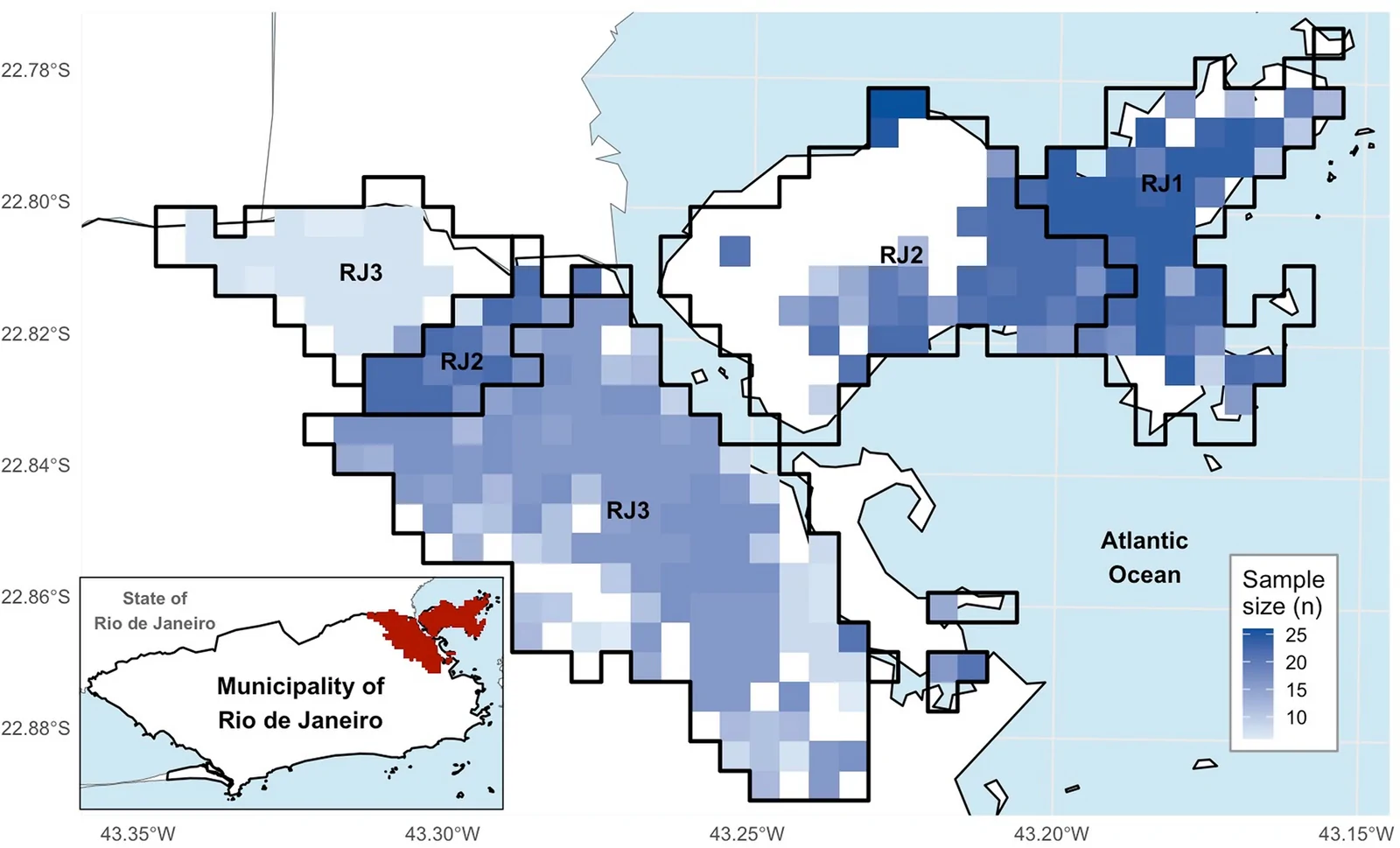
Map of raster cell sample sizes by *Wolbachia* release zone. Sample size represents the number of months of *Wolbachia* prevalence data available across the total release period. The black outline indicates the total release area (*n* = 396). Raster cells with no *w*Mel data (*n* = 109) or fewer than 5 months of data (*n* = 20) were excluded, leaving 267 raster cells (blue) included in the analysis. The three release zones are labelled RJ1 (releases from August 2017), RJ2 (November 2017), and RJ3 (February 2018, August 2018, and February 2019). The inset shows the municipality of Rio de Janeiro, with the release area highlighted in red

For each raster cell, monthly *w*Mel prevalence was calculated as the proportion of tested *Ae. aegypti* positive for *w*Mel. Raster cells with no *w*Mel data (*n* = 109) or fewer than 5 months of observations (*n* = 20) were excluded, leaving 267 cells (Fig. 1). To standardise heterogeneous follow-up across release areas, a binomial generalised linear model was fitted to each cell and used to generate smoothed 26-month wMel prevalence trajectories from first release. Monthly mean temperature was included to account for irregular sampling and heterogeneous temporal coverage and was not interpreted as an explanatory variable in this step. K-means clustering was then applied to these predicted trajectories to define the categorical outcome used in the environmental regression model. Full details of trajectory derivation are provided in Additional file 1.

### Covariates: climate and landscape features

A selection of 23 spatially explicit climate and landscape covariates were compiled, guided by ecological mechanisms influencing the *Ae. aegypti* and *Wolbachia* dynamics. Specifically, covariates focussed on *Ae. aegypti* habitat suitability, climate sensitivity, flight barriers, and *Wolbachia* deployment barriers (Additional file 1: Table S2). Examples from each respective category include human population and vegetation cover, elevation and temperature, proportion of highways and the proportion of non-residential buildings, the proportion of favelas and average household income.

Covariate data were obtained from publicly available sources where possible, harmonised to the 500 × 500 m grid using area-weighted areal interpolation for partially overlapping polygons and rasters, point-in-polygon summation for point features, and inverse distance-weighted interpolation for climate variables. Covariates related to mosquito dispersal (Additional file 1: Table S2, category: *Ae. aegypti* flight barriers) were extracted within each cell and a surrounding 200 m buffer. Covariates with zero variance were excluded. Highly correlated covariates (coefficients >|0.8|) were screened and excluded or transformed as appropriate, driven by ecological interpretability and retaining covariates which are most informative determinants of *Ae. aegypti* distribution. Full details of covariate harmonisation and screening are provided in Additional file 1.

Environmental covariates were included as linear terms rather than smooth functions to maintain model interpretability and avoid overfitting given the relatively small sample size (*n* = 267). Preliminary exploration indicated largely monotonic relationships with replacement outcomes, and therefore, non-linear smoothing was applied only to the spatial autocorrelation term. Although covariate effects were modelled linearly on the multinomial logit scale, predicted trajectory probabilities may still exhibit non-linear dynamics due to the multinomial model structure and spatial smooth term.

### Model selection

To identify environmental and landscape factors associated with different *w*Mel replacement trajectories, the categorical outcome of cluster assignment was modelled using a GAM in the mgcv R package [60]. Model diagnostics followed standard GAM evaluation procedures including basis dimension diagnostics and residual inspection. GAMs are a common way to model complex and potentially non-linear associations between climate and landscape factors and mosquito ecology [61–65]. Since *Wolbachia* dynamics are mediated through their mosquito hosts, this approach is appropriate for modelling *Wolbachia* replacement and can accommodate spatial autocorrelation.

Forward selection was performed, initially selecting the covariate with greatest reduction in Akaike Information Criterion (AIC) among univariate models and repeating this step to select additional covariates using a stepwise approach until no additional covariates reduce AIC by > 2 [66, 67 ]. Covariate selection was performed prior to including a spatial smooth so that covariates competed without their effects being absorbed by the spatial term [68, 69 ]. While this approach cannot fully eliminate spatial confounding, it reduces the risk that flexible spatial effects dominate model selection and mask associations with environmental covariates. We considered incorporating spatial terms during model selection as an alternative approach; however, given the limited sample size and spatial resolution, this can lead to unstable estimates when spatial smooths compete with covariates.

Four covariates were selected for inclusion in the model: the proportion of tall buildings (% > 6 m in height), average air temperature (°C), average elevation (m above sea level), and average daily rainfall (mm). We modelled the four-level replacement trajectory outcome $\widehat{{Y}_{c}}$, defined as the cluster-assigned trajectory category for each raster cell c, using a multinomial GAM. Let ${P}_{ck}=\mathrm{P}\mathrm{r}(\widehat{{Y}_{c}}=k)$ denote the probability that raster cell c belongs to replacement trajectory $k\in \{1, 2, 3, 4\}$, with 1 as baseline. The unsuccessful replacement trajectory was assigned as the reference baseline, since any of the three success trajectories demonstrate some degree of progress towards *Wolbachia* replacement goals. For each of the other trajectories, k=2,3,4, the log-odds were modelled as

$$\log \,\left( {\frac{{P_{ck} }}{{P_{c1} }}} \right)\, = \,\alpha_{k} \, + \,\beta_{1k} tall\_build_{c} \, + \,\beta_{2k} temp_{c} \, + \,\beta_{3k} elev_{c} \, + \,\beta_{4k} rain_{c} \, + \,f_{k,z\left( c \right)} \,\left( {wMel_{c} } \right)$$

where ${tall\_build}_{c}$, ${temp}_{c}$, ${elev}_{c}$, and ${rain}_{c}$ denote, respectively, the proportion of tall buildings (%), average air temperature (°C), average elevation (m above sea level), and average daily rainfall (mm) in raster cell c. ${\alpha}_{k}$ is the trajectory-specific intercept, and ${\beta}_{jk}$ are the trajectory-specific regression coefficients for each linear covariate j. The multinomial probabilities were obtained using ${P}_{ck}=\mathrm{e}\mathrm{x}\mathrm{p}({\eta}_{ck})/{\sum}_{m=1}^{4}\mathrm{e}\mathrm{x}\mathrm{p}({\eta}_{cm})$, where ${\eta}_{ck}=\mathrm{l}\mathrm{o}\mathrm{g}({P}_{ck}/{P}_{c1})$ for k=2,3,4 and ${\eta}_{c1}=0$. All covariates were defined at the raster cell level to match the spatial structure of the input data. For each covariate, we derived partial-dependence (PD) curves by trajectory. Local effects were computed as the relative change in predicted probability per trajectory for a specified unit increase in the covariate (finite differences along the PD curve).

Due to strong evidence of spatial autocorrelation, we included a smoothed spatial lag term by release zone to account for local spatial dependence rather than as a predictor of ecological interest (Fig. 1; RJ1, RJ2, RJ3). For each raster cell c, the average proportion of *w*Mel-infected *Ae. aegypti* was estimated in all neighbouring raster cells excluding the focal cell, ${wMel}_{c}$, producing a 500–700 m spatial lag (accounting for diagonal cell distance of queen neighbours). Partial dependency plots of the average neighbouring proportion of *w*Mel show the predicted probability of a raster cell being assigned to a replacement trajectory varied across the three release zones. Thus, ${f}_{k, z(c)}\left(\bullet \right)$ is a smooth function of ${wMel}_{c}$ estimated using a thin-plate regression spline (bs = ”ts”, *k* = 10) for each release zone, z. Smoothness was selected automatically through the mgcv penalisation framework, allowing unnecessary smooth terms to be shrunk towards zero.

## Results

**Fig. 2:**
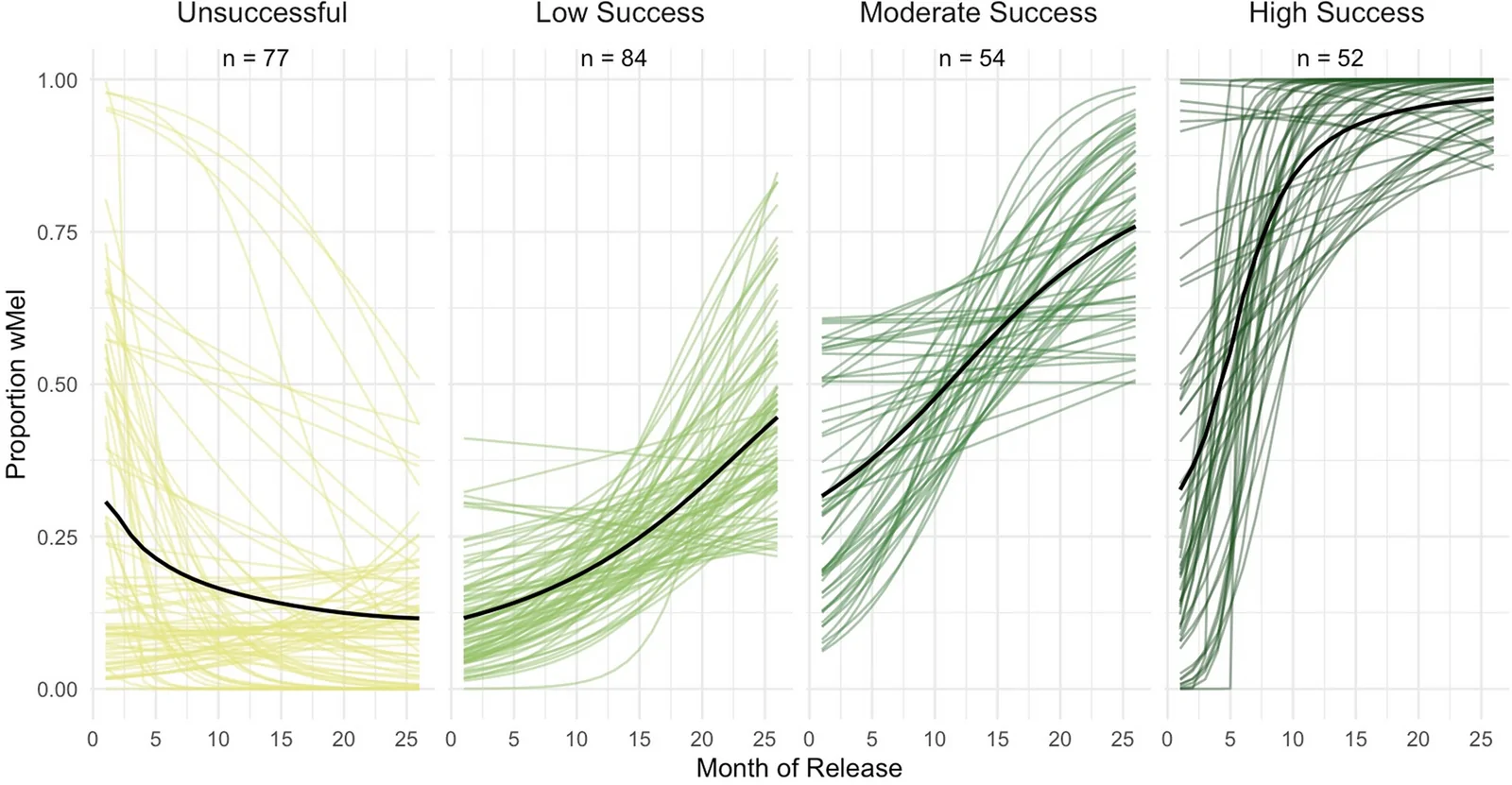
Model-predicted trajectories of *w*Mel proportion over the release period. Lines show the predicted *w*Mel proportion for each raster cell assigned to each replacement trajectory, with black lines indicating the mean trajectory. Time is expressed as months since the first *Wolbachia* release within each raster cell

The 267 raster cells were first clustered into four replacement trajectory groups: unsuccessful (*n* = 77, 28.8%), low (*n* = 84, 31.5%), moderate (*n* = 54, 20.2%), and high success (*n* = 52, 19.5%). Individual raster cell trajectories in any of the low, moderate, or high success groups with a borderline declining slope coefficient (< −0.005) were manually reassigned to the unsuccessful trajectory (moderate success: *n* = 7; high success: *n* = 2; Additional file 1: Fig. S3). The unsuccessful trajectory was characterised by model-predicted *Wolbachia* prevalence decreasing from 31 to < 20% within 6 months on average, and then declining more slowly reaching a mean of 12% by the end of the release period (Fig. 2). The low- and moderate-success trajectories increased steadily from 12 to 45% and from 32 to 76% *Wolbachia* prevalence, respectively, whereas the average trend for the high-success trajectory was rapid increase in model-predicted *Wolbachia* prevalence from 33 to > 90% within 13 months on average, eventually stabilising at 96%. Some highly successful raster cells showed little overall change in model-predicted *Wolbachia* prevalence, remaining > 80% throughout the 26-month release period from initial release.

**Fig. 3:**
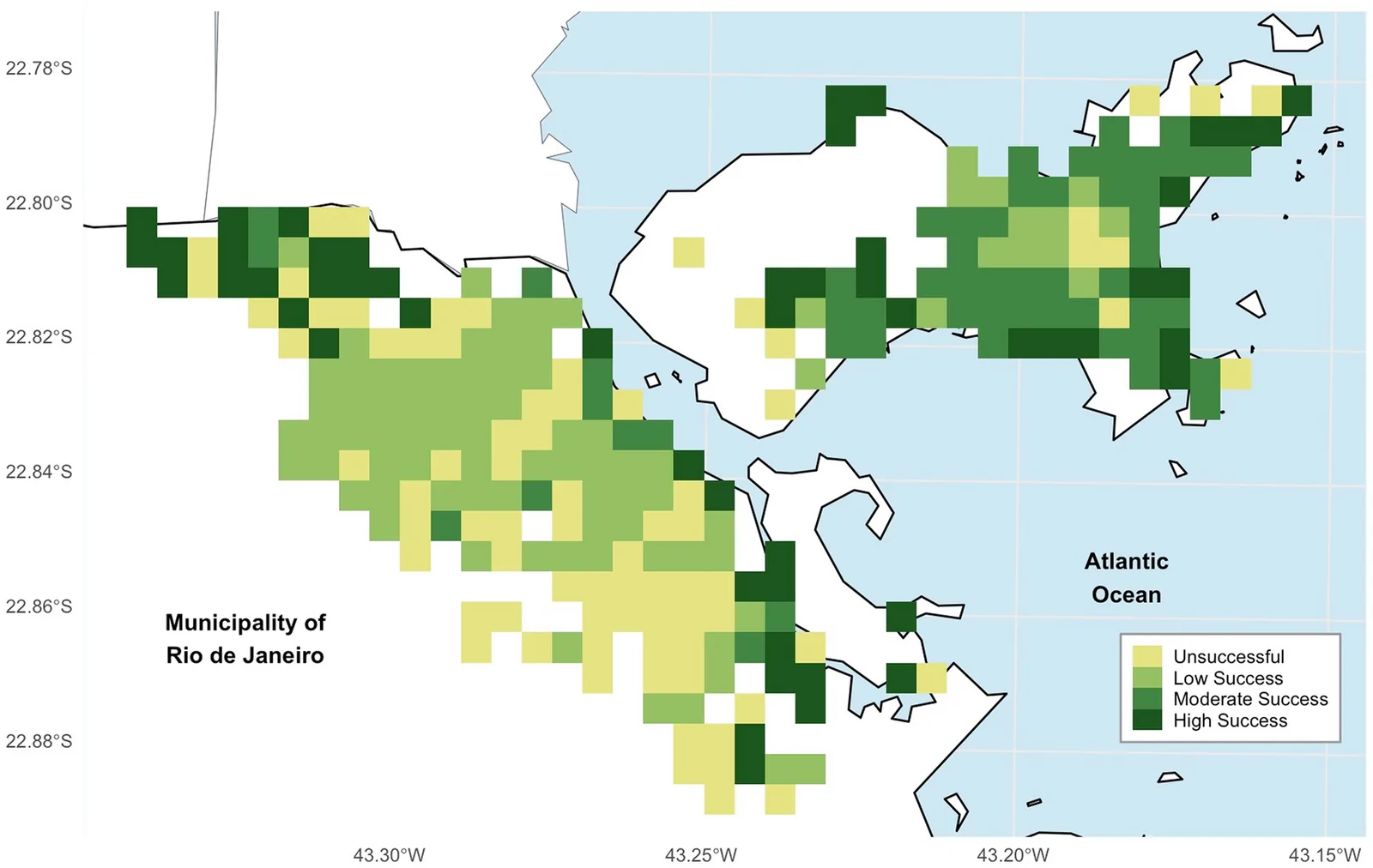
Spatial distribution of raster cells according to replacement trajectory. Colours indicate the replacement trajectory assigned to each raster cell

Four covariates were selected for inclusion in the model: the proportion of tall buildings (% > 6 m in height), average air temperature (°C), average elevation (m above sea level), and average daily rainfall (mm). The proportion of tall buildings is a covariate from the category *Ae. aegypti* flight barriers, while the latter three covariates are from the climate sensitivity and breeding habitat covariate categories. To assess the influence of manual trajectory reassignment (*n* = 9), we repeated model selection without trajectory reassignment. The results remained broadly consistent, with the proportion of tall buildings and temperature selected as primary covariates, while some secondary covariates showed minor differences in selection and effect size. Under average environmental and landscape conditions across the release area, the multinomial GAM predicts a baseline probability of 23.9% for unsuccessful replacement, 47.7% for low-success, 22.7% for moderate-success, and 5.7% for high-success trajectories. These were estimated per release zone using zone-specific median covariate values including the spatial lag term, and overall values obtained by weighting zone-level predictions by the number of raster cells. However, there are some notable spatial trends. Monte Carlo Join-Count tests for each group showed strong evidence of spatial autocorrelation (FDR-adjusted *P* < 0.01), showing that replacement trajectories are spatially clustered (Fig. 3) which may also reflect differences in release timing and deployment intensity across zones.

A higher proportion of raster cells show moderate or high success on Ilha do Governador, an island with Rio de Janeiro international airport on the Northwest side (64 of 90, 71.1%), compared to the mainland (42 of 177, 23.7%). Similarly, under median environmental and landscape conditions within release zones, the probability of an area having moderate or high success (61.8% and 12.9%, respectively) is higher for release zone 1 (RJ1), the East side of Ilha do Governador, compared to the other release zones (Table 1). This is consistent with these areas on Ilha do Governador being the first release sites, having more months of releases in total (RJ1 sample median 23, range 9–24, and majority of RJ2 median 21, range 7–26; Fig. 1) before releases expanded West and to the mainland (RJ3 sample median 15, range 6–21). However, inclusion of release zone as a fixed effect in a sensitivity analysis did not improve model fit and did not materially alter the estimated effects of the retained environmental covariates, suggesting that broad differences in release timing and programme duration between zones do not fully explain the observed replacement trajectories. On the mainland, a general trend can be observed where raster cells with moderate or high success tend to be closer to the coastline than inland (Fig. 3) which may be associated with the region’s elevation profile, discussed further in the elevation subheading.

**Table 1:** Median covariate values and baseline model-predicted probability of *Wolbachia* replacement trajectories by release zones (RJ1, RJ2, RJ3)

	RJ1	RJ2	RJ3
Median covariate values			
Proportion of tall buildings (% > 6 m)	0.392	0.400	0.499
Mean air temperature (°C)	24.5	24.5	24.6
Elevation (m)	12.6	12.5	9.11
Mean daily rainfall (mm)	3.58	3.50	3.43
Mean proportion neighbouring *w*Mel (%)	0.347	0.183	0.0894
Baseline model-predicted probability of *Wolbachia* replacement trajectories			
Unsuccessful	10.2%	20.0%	28.5%
Low success	15.1%	40.1%	58.4%
Moderate success	61.8%	35.6%	9.1%
High success	12.9%	4.4%	4.0%

RJ1 is the area on the East side of Ilha do Governador (sample median 23, range 9–24); RJ2 is the area on the West side of Ilha do Governador and expanding onto mainland (sample median 21, range 7–26); RJ3 is the majority of mainland release areas (sample median 15, range 6–21); see Fig. 1 for mapped areas

### Tall buildings

**Fig. 4:**
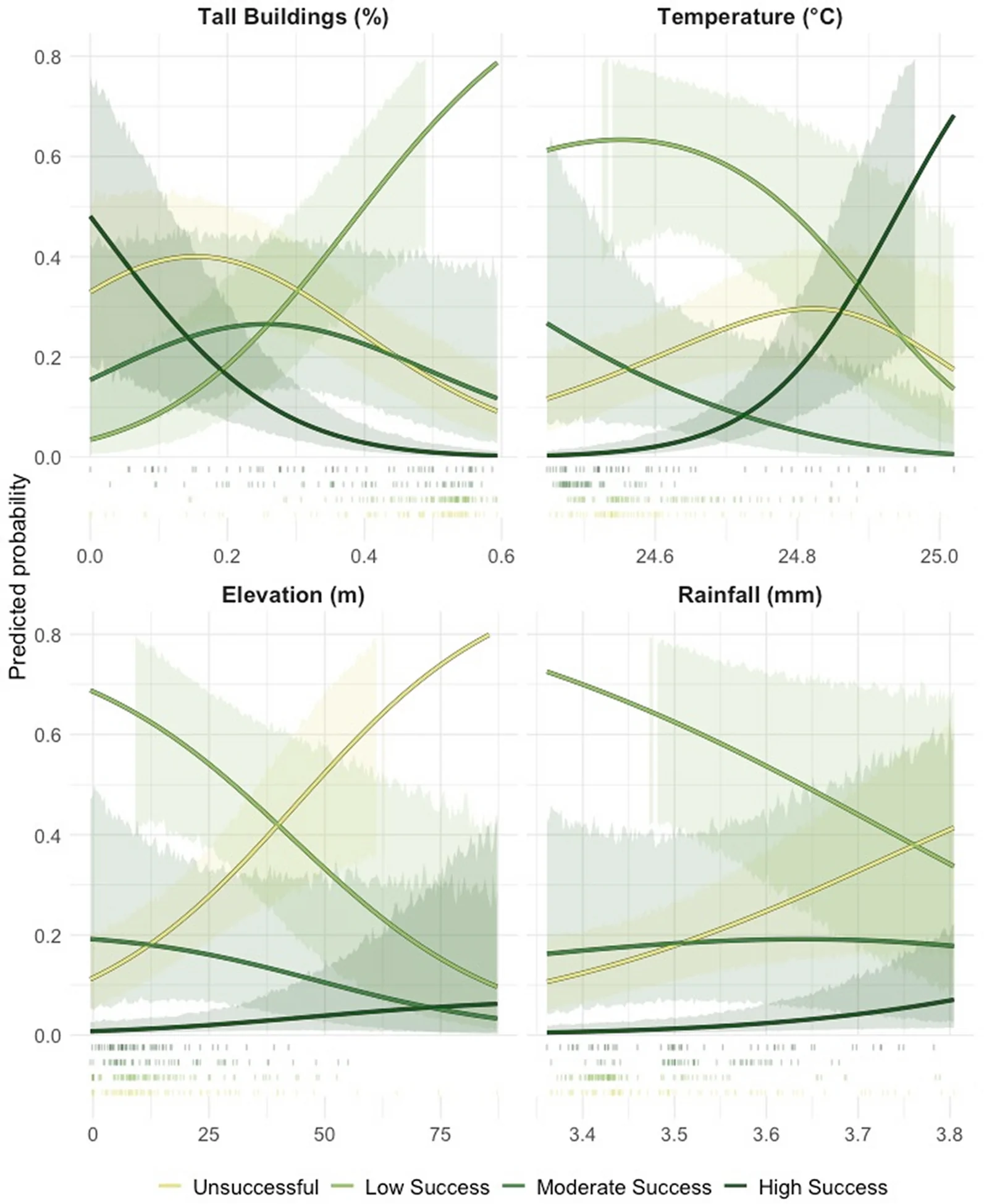
Partial dependence plots showing the predicted probability of each replacement trajectory across selected model covariates. Panels show the four covariates retained in the final multinomial generalised additive model (GAM): proportion of tall buildings (> 6 m in height), mean air temperature (°C), mean elevation (m), and mean daily rainfall (mm). Coloured lines show the predicted probability of each replacement trajectory. Predictions were generated by varying the focal covariate across its observed range while holding all other continuous covariates, including the spatial smooth term, at their median values and categorical covariates at their reference level. Shaded ribbons represent approximate 95% confidence intervals (CI) derived from the fitted model prediction standard errors. Rug plots beneath each panel indicate the observed distribution of covariate values for each replacement trajectory

The proportion of tall buildings (% > 6 m in height) was the first covariate selected by the multinomial GAM, ranging from 0.0 to 59.4% (median 47.9%) across the release area (Additional file 1: Fig. S4). High replacement success was strongly associated with a lower proportion of tall buildings [*β* = −0.93 (95% CI −1.43, −0.44), *P* < 0.001], whereas low replacement success was strongly associated with a higher proportion of tall buildings [*β* = 1.10 (95% CI 0.57, 1.63), *P* < 0.001]. Using model-based predictions, a + 10% increase from the median proportion of tall buildings was associated with a 13.5% increase in the probability of low replacement success and reduced probability of all other trajectories (Fig. 4). Sensitivity analyses re-ran model selection sequentially excluding each trajectory, consistently showing that areas with a higher proportion of tall buildings were more likely to experience low replacement success, while areas with fewer tall buildings are more likely to achieve high replacement success.

To investigate the degree of building height associated with *Wolbachia* replacement success, another sensitivity analysis redefined tall buildings as > 15 m in height rather than > 6 m (the next available height category in the dataset; Additional file 1: Table S2). Under this definition, the proportion of tall buildings was no longer strongly colinear with building volume, allowing building volume to be retained during model selection rather than excluded as in the primary analysis. In this sensitivity analysis, building volume was selected first, with the remaining covariates selected in the same order (average air temperature, average elevation, and average daily rainfall). This suggests that when tall buildings are defined at a lower height threshold (> 6 m), the proportion of tall buildings captures much of the same underlying urban structural variation as building volume. While the first selected covariate differs, the findings concur that high urban density and vertical structure (whether measured by height or volume) present a challenge for *Wolbachia* replacement.

Urban structure was also captured by the proportion of vegetation cover, which was the next best performing covariate after rainfall; however, it was not selected as ΔAIC <|2|. When forced in the model, the strength of covariate associations did not noticeably change except within the high success trajectory. In this analysis, high replacement success was significantly associated with a higher proportion of vegetation cover [*β* = 0.61 (95% CI 0.09, 1.12), *P* = 0.020], and no longer strongly associated with lower proportions of tall buildings [*β* = −0.55 (95% CI −1.15, 0.05), *P* = 0.071]. Suggesting vegetation cover and vertical urban structure may be capturing shared underlying urban characteristics associated with high replacement success trajectory in this sensitivity analysis.

### Temperature

Average air temperature (°C) (mean across 26-month release period) was the second covariate selected by the multinomial GAM, ranging from 24.5 to 25.0 °C (median 24.6 °C) (Additional file 1: Fig. S5). High replacement success was strongly associated with higher temperatures within the observed local range [*β* = 1.11 (95% CI 0.59, 1.62), *P* < 0.001], a result which was maintained across all sensitivity analyses in which the model was re-run without each trajectory. In the primary analysis, lower temperatures were also strongly associated with moderate replacement success [*β* = −0.94 (95% CI −1.81, −0.07), *P* < 0.05], although this result was not maintained through sensitivity analyses.

Using model-based predictions, a + 0.2 °C increase in average air temperature to 24.8 °C is associated with an 11.1% increased probability of *w*Mel replacement failure (unsuccessful). This temperature increase is associated with reduced probability of low replacement success (−9.5%) and moderate success (−11.2%), yet increased probability of high success (9.6%). Notably, the predictions suggest divergent outcomes, where higher temperatures increase the probability of both high replacement success and replacement failure. The consistency of this bistable trend across temperature and the proportion of tall buildings may be linked to urban heat island effects which have a higher incidence of dengue than other urban areas [70–73].

### Elevation

Average elevation (m above sea level) was the third covariate selected by the multinomial GAM. Lower elevation was strongly associated with low ([*β* = −0.63 (95% CI −1.04, −0.23), *P* < 0.01] and moderate replacement success [*β* = −0.59 (95% CI −1.12, −0.08), *P* = 0.024]. However, elevation ranged from −0.60 to 87.21 m (median 10.86 m) across the release area (Additional file 1: Fig. S5). *Ae. aegypti* are found at high altitude [74, 75 ], and there is no known biological limitation to *Wolbachia* at elevation; thus, confounding is likely, i.e. the effect of elevation identified by the model captures underlying variation which was not captured in the available covariates or the spatial smooth term. To explore this further, several sensitivity analyses were conducted.

One hypothesis is the higher likelihood of favelas on higher or steeper ground due to the prohibitive cost of building formally organised housing and infrastructure. However, the variable proportion of favelas was not selected by the model. First, we forced the proportion of favelas covariate to be included in the final model which had small positive effect sizes across all trajectories, none significant [*β* = 0.11 (95% CI −0.23, 0.45), *β* = 0.14 (95% CI −0.30, 0.59), *β* = 0.10 (95% CI −0.30, 0.49); low, moderate and high success, respectively]. Elevation effect sizes became marginally smaller, yet overall, no significant changes were observed because of this forced inclusion. Second, we noticed a spatial trend of higher proportion of favelas being clustered either at higher elevation or near the coast, at low elevation. Thus, the proportion of favelas was split by the median elevation into two variables: the proportion of favelas at high and low elevation. Model selection was re-run and there was no change in the covariate selection, i.e. neither favela variable was selected and the elevation variable was still selected. This suggests the proportion of favelas independently and the intersection of favelas at high elevation do not explain this result.

Additionally, changes in urban topology across elevation gradients likely impacted the logistical challenges of deployment. While we do not have access to publicly available data of how many mosquitoes were released in which areas, this could be a key contributor to observed variation in *Wolbachia* replacement trajectories at different elevation. Since elevation may be linked to increased slope gradients, increasing elevation could impact *Ae. aegypti* population dynamics through changes in wind exposure, solar exposure and water runoff, which impacts the effects of rainfall on breeding habitats. Regardless of the underlying mechanism, higher elevation was consistently strongly associated with low replacement success across sensitivity analyses in which the model was re-run without each trajectory. Additionally, model-based predictions suggest that higher elevation is associated with an increased probability of an area having an unsuccessful *w*Mel replacement trajectory (Fig. 4). A + 10 m increase in elevation draws *w*Mel replacement trajectories away from low replacement success (−6.4%) and is associated with a 7.3% increased probability of replacement failure (unsuccessful trajectory). Collectively these findings suggest an association between higher elevation and lower likelihood of successful *Wolbachia* replacement in the municipality of Rio de Janeiro, although the underlying mechanisms remain unresolved.

### Rainfall

Average daily rainfall (mm) was the fourth and final covariate selected by the multinomial GAM, ranging from 3.36 to 3.80 mm (median 3.48 mm) (Additional file 1: Fig. S5). Lower rainfall was strongly associated with increased risk of low replacement success [*β* = −0.49 (95% CI −0.93, −0.06), *P* = 0.027] in the primary analysis. Using model-based predictions, a + 0.25 mm increase (median 3.73 mm) shifts *w*Mel replacement trajectories away from low replacement success (probability reduced by 23.3%) while increasing the probability of unsuccessful replacement by 18.9%. The probabilities of moderate and high replacement success change only minimally (+ 0.6% and 3.9%, respectively), indicating a redistribution from low success towards the extremes, especially replacement failure (Fig. 4). Rainfall is a known ecological driver of *Ae. aegypti* breeding site availability, although its effects are context dependent. However, rainfall was not consistently selected in sensitivity analyses when the model was re-run with individual trajectories excluded, suggesting that its effect on *w*Mel replacement specifically has the weakest support among the four covariates.

### Model performance

The multinomial GAM explained 25.3% of the variation in replacement trajectories (percent deviance explained), including the spatial smooth term for neighbouring *w*Mel prevalence. Simpler models including only the spatial smooth term explained substantially less variation (12.8%), indicating that environmental covariates contributed meaningful explanatory power. Model diagnostics indicated satisfactory model performance as the model converged fully with stable parameter estimation. Basis dimension checks showed no evidence that the spatial smoothing parameter k was insufficient (all k-index ≈0.95, *P* > 0.20) and the estimated degrees of freedom for the spatial smooth ranged from 3 × 10^–5^ to 1.01 across trajectories, indicating predominantly linear or negligible spatial effects and confirming that the shrinkage spline effectively prevented overfitting [60].

The confusion matrix indicated an overall correct classification rate of trajectory of 0.58 (95% CI 0.52–0.64). Model sensitivity was highest for moderate success and low replacement success (0.70 and 0.65, respectively) and moderate for high success (0.50) and the unsuccessful trajectory (0.47). Specificity exceeded 0.80 for all trajectories, indicating the model was conservative in classification even where sensitivity was limited. However, nearly half of truly unsuccessful cases were misclassified, most frequently as low or high success. This indicates that the model is less reliable for identifying unsuccessful trajectories compared with other trajectories, reflecting trajectory-specific performance differences despite reasonable overall classification accuracy.

Spatial smooth terms for average neighbouring proportion of *w*Mel varied across replacement trajectories. The spatial term was strongly significant for moderate-success trajectories across all release zones (RJ1 *P* < 0.01; RJ2 and RJ3 *P* < 0.001), high-success trajectories across release zones 1 and 2 (RJ1 and RJ2 *P* < 0.001), and low-success trajectories in release zone 3 (RJ3 *P* < 0.001). This indicates the relationship between average neighbouring proportion of *w*Mel and replacement trajectory is dependent on release zone. Analysis of spatial autocorrelation suggested residual spatial structure remained for some trajectories. Moran’s I was significant for unsuccessful (*I* = 0.070, *P* = 0.034), low success (*I* = 0.145, *P* = 0.001), and high success (*I* = 0.075, *P* = 0.018), indicating mild spatial clustering of residuals and implying some unexplained spatial structure persists.

## Discussion

This study examined how climate and landscape features shape spatially heterogeneous *Wolbachia* replacement in a large-scale urban deployment in the municipality of Rio de Janeiro. By clustering *w*Mel replacement trajectories and modelling their associations with environmental factors, we identified four covariates associated with replacement success: lower proportion of tall buildings (% > 6 m in height), higher air temperature (°C), lower elevation (m above sea level), and higher daily rainfall (mm). Together, these results highlight the importance of urban structure and environmental conditions that influence mosquito dispersal, population connectivity, and life history traits underpinning *Wolbachia* dynamics.

Urban structure, particularly the vertical built environment, emerged as the strongest and most consistent covariate associated with replacement trajectories. Areas with a higher proportion of tall buildings were more likely to exhibit low replacement success, whereas areas with fewer tall buildings were more likely to achieve high success. High-rise clusters can function as semi-permeable barriers to horizontal mosquito dispersal through shading, wind channels, and canyon effects [62, 76, 78 –], and individual buildings represent opportunities for vertical dispersal into distinct habitats [76, 79, 80 ], potentially reducing effective mixing between released and wild *Ae. aegypti*. One hypothesis is that high-rise buildings create a trade-off in which reduced immigration of uninfected mosquitoes from surrounding areas may facilitate *Wolbachia* invasion by reducing dilution of *Wolbachia* [58], while simultaneously constraining the conditions required for high-level, stable establishment. However, the mechanism remains unknown as tall buildings may also represent urban density, socioeconomic conditions, or release accessibility. Evidence from other *Wolbachia* releases supports how building structure can shape local mosquito dynamics at a fine scale. For example, in Malaysia, *w*AlbB-infected *Ae. aegypti* established across a high-rise residential site with an infection prevalence > 90% within 12 weeks and remained high for at least 5 years [80]. Of the eight residential blocks, some were invaded more rapidly than others and *Wolbachia* frequencies were found to be highest on the 2nd and 8th floors (the middle floor).

Previous metapopulation models predict that *Wolbachia* replacement outcomes depend on local mosquito migration rates and release density [81, 82 ]. Fine-scale release density data were unavailable for this analysis, and therefore, residual confounding by deployment intensity remains a limitation. Although the data source indicates that releases occurred over extended periods and in multiple phases across the study area [22], adjustment for release zone in a sensitivity analysis produced similar coefficient estimates and did not improve model fit, suggesting that broad differences in release timing and programme duration between zones are unlikely to explain the observed associations. However, release intensity could not be quantified at the spatial resolution of the replacement trajectories analysed here. Consequently, the environmental associations identified should be interpreted as hypothesis-generating and may partially reflect unmeasured variation in deployment intensity or other operational factors. Additionally, host fitness costs linked to insecticide resistance identified in early Rio de Janeiro pilot releases [59] were addressed operationally prior to the releases analysed here, suggesting that observed heterogeneity is unlikely to be driven solely by host fitness trade-offs, although variation in fitness likely remains.

Sensitivity analyses re-defining tall buildings as > 15 m revealed that building volume became the dominant covariate, indicating that *Wolbachia* success is influenced by broader urban topology rather than building height alone. Vegetation cover, although not selected in the primary analysis, was significantly associated with high replacement success when forced into the model and displaced the importance of tall buildings for this trajectory. This suggests that while tall buildings are the stronger overall covariate distinguishing replacement trajectories, vegetation may reflect habitat suitability factors that could be associated with high-level *Wolbachia* establishment, such as shaded microclimates, resting sites, and stable breeding conditions.

Climate variables further shaped replacement trajectories. Higher temperatures within the observed local range increased the probability of both high replacement success and complete failure while reducing the probability of intermediate trajectories (Fig. 4). This pattern aligns with theoretical expectations that environmental stress can amplify population instability, pushing *Wolbachia* dynamics towards divergent outcomes [83–89], and with threshold-dependent invasion models in which outcomes are governed by unstable internal equilibria and bistability [38, 58, 90 ]. Mechanistically, these results may reflect a trade-off between the benefits of higher temperatures for *Ae. aegypti*, such as faster development, higher survival and increased fecundity [91–96], and the costs to *Wolbachia,* where cyclical temperature regimes (52,54; 26–37 °C, 23.7–35.3 °C, respectively) or heatwaves [47, 49 ] can reduce *Wolbachia* infection density or even trigger infection loss [97, 98 ]. In invasion models, crossing critical thresholds can lead to rapid transitions between persistence and loss of infection. However, this analysis is observational and does not directly identify dynamical attractors or invasion thresholds. The apparent bistability arises from non-linear structure in the fitted multinomial GAM partial-dependence surfaces and should be interpreted as model-based prediction of heterogeneous responses requiring experimental validation.

Elevation was consistently associated with low replacement success and model-predicted probabilities suggested higher elevation is associated with unsuccessful replacement, despite a lack of known biological constraints on *Ae. aegypti* or *Wolbachia* at the elevations observed. This suggests elevation may act as a proxy for unmeasured environmental, infrastructural, socioeconomic, or operational gradients that vary across the urban landscape. A plausible operational explanation may be that favelas present implementation and surveillance challenges which covary with elevation; however, sensitivity analyses exploring the role of hillside favelas did not explain this association. Other factors that may covary with elevation, including differences in vegetation, drainage, road accessibility, housing characteristics, or water storage behaviours, were not directly assessed. Regardless of mechanism, the consistency of this result indicates that elevation-related urban features in the municipality of Rio de Janeiro warrant further investigation to refine release strategies in complex urban landscapes.

Rainfall influenced replacement trajectories by redistributing probability towards low-success trajectories and replacement failure. This broadly aligns with the role of rainfall in generating breeding sites and flushing of immature mosquito stages [39, 40, 99 ]. However, mechanistic processes were not directly assessed, and rainfall was inconsistently selected across sensitivity analyses, indicating weaker support for its role relative to other covariates.

Mild residual spatial clustering persisted even after incorporating spatial smooth terms, suggesting that neighbourhood-scale processes, including local urban structure, operational deployment intensity and behavioural factors, continue to influence replacement outcomes. Although the overall deviance explained by the model was moderate (25.3%), this is expected in complex urban ecological systems where many operational and behavioural factors remain unmeasured. Classification performance was also moderate, particularly for unsuccessful and high-success trajectories. However, the primary objective of the modelling framework was explanatory rather than predictive, and the identified covariates were associated with gradual shifts in the probability of replacement trajectories. A GAM was selected over alternative machine learning approaches, such as regression trees or random forests, because they provide interpretable estimates of covariate effects while allowing flexible spatial structure [60]. This supports our goal of explanatory inference rather than maximising predictive accuracy. Reliance on publicly available datasets imposed several limitations, for example, aggregation to 500 × 500 m cells, lack of release density data, and reliance on interpolated covariates. In addition, several sociodemographic covariates were derived from the 2022 census, and several environmental covariates were derived from remotely sensed imagery taken between 2019 and 2022, whereas releases occurred between 2017 and 2019. Although many urban landscape characteristics are relatively stable over this timescale, temporal mismatch may have introduced measurement error for more dynamic variables such as population density, income, and sanitation conditions. Potential limitations also include residual spatial confounding, collinearity among covariates despite screening, and uncertainty associated with the forward AIC model-selection procedure. Although the bootstrap stability analysis demonstrated that the selected covariates were highly consistent across resampled datasets (see Additional file 1), the forward selection procedure evaluated 19 candidate covariates without formal adjustment for multiple testing; thus, the possibility of false-positive covariate selection cannot be excluded. Future studies integrating direct measures of release effort and higher-resolution environmental data could help refine the model and further clarify the drivers of heterogeneous *Wolbachia* replacement.

From an operational perspective, areas with high-rise buildings and topographically complex areas showed more divergent and unpredictable outcomes, sensitive to small perturbations and less likely to stabilise at intermediate replacement. Incorporating urban permeability and vertical structure into predictive models could help identify high-risk areas prior to deployment and inform operational decisions such as increasing release density, modifying release strategies (e.g. targeting individual buildings or floors), or allocating additional monitoring resources. As programmes expand across heterogeneous urban landscapes, replacement data from early deployments can be incorporated into updated models to identify emerging barriers to *Wolbachia* establishment, such as environments where urban structure limits mosquito or *Wolbachia* spread. In this way, modelling and field deployment can operate in a feedback loop in which empirical observations inform model refinement and models in turn guide adaptive adjustments to release strategies and monitoring.

Overall, this study demonstrates that publicly available climate and landscape datasets can generate operationally relevant hypotheses about the drivers of heterogeneous *Wolbachia* replacement. In complex disease systems such as dengue, integrating environmental data with empirical replacement trajectories is a compelling approach for identifying mechanisms underlying spatial variation in intervention outcomes. These findings may inform future field studies and adaptive deployment strategies aimed at improving *Wolbachia* establishment.

## Conclusions

Areas characterised by a higher proportion of tall buildings were more prone to divergent *Wolbachia* replacement dynamics in the municipality of Rio de Janeiro, with model-based predictions indicating temperature was associated with increased probabilities of both high success and failure. Incorporating urban permeability and vertical structure into predictive models may help identify areas with greater uncertainty in replacement outcomes prior to deployment and could support operational planning, including considerations such as release density, modifying release strategies (e.g. targeting individual buildings or floors), or prioritising monitoring resources. This work provides a basis for further iterative modelling and field evaluation, where modelling and deployment activities can operate in a feedback loop to better understand barriers to *Wolbachia* establishment.

## Supplementary Information

Additional file 1.

## Data Availability

The codebase and associated datasets used in this study are available in the GitHub repository https://github.com/katietiley/wMel_environ_associations_Rio. The *Wolbachia* prevalence dataset analysed during the current study are available in the GitHub repository https://github.com/pdgcam/wMel_Rio_dengue_chik [22].
