# Supplementary material for: Climate and landscape features associated with successful Wolbachia replacement in the municipality of Rio de Janeiro, Brazil
Source: Parasit Vectors. 2026 Aug 4;19:469. doi: 10.1186/s13071-026-07602-y (PMC13726168; doi:10.1186/s13071-026-07602-y)
Supplement: Supplementary file 1 [file 13071_2026_7602_MOESM1_ESM.docx]

**Supplementary** **Materials to ‘Climate and landscape features associated with successful *Wolbachia* replacement in the municipality of Rio de Janeiro, Brazil**’

**Authors:** Katie Tiley, Katie Milligan Susong, Steffen Knoblauch, Kathleen M O’Reilly, and Oliver Brady

**Expanded Methods**

*Outcome: wMel replacement trajectories*

For each BG-Sentinel trap collection, up to ten *Ae. aegypti* (male and female) were individually tested for wMel by qualitative PCR [1]. Since the number of traps and tested mosquitoes varied across space and time, the main outcome variable was defined as the proportion of wMel-positive mosquitoes per raster cell per month. Raster cells with no wMel data were removed (n=109). The release area expanded over time, thus the maximum months of data for different areas varied (8–26 months). Within different release areas individual raster cells had incomplete data due to traps not being placed at all locations at all times, thus raster cells with less than five months of data were excluded (n=20) resulting in average missingness per raster cell of 20.0% (0.0–69.6%). To define replacement trajectories across a standardised release period, first a binomial generalised linear model (GLM) with logit link was fitted separately for each raster cell (n=267), predicting wMel proportions across 26 months from first release in each raster cell. The GLM was specified as follows:

$$y_{ct}\sim Binomial\left( n_{ct},\mu_{ct} \right)$$

$$logit\left( \mu_{ct} \right)=\beta_{0}+\beta_{1}{month}_{ct}+\beta_{2}{temp}_{ct},$$

where $y_{ct}$ denotes the proportion of wMel-infected *Ae. aegypti* in raster cell $c$ at month $t$, $n_{ct}$ is the number of mosquitoes tested (up to ten per trap collection, summed across collections for each raster cell-month unit), and $\mu_{ct}$ is the modelled infection probability. The GLM included month of release, $month$, and monthly mean temperature ($℃$; inverse distance weighted from meteorological stations), $temp$, as covariates. After fitting this model, predictions of wMel prevalence trajectories, $\hat{Y_{c}}$, adjusted for mean temperature within raster cell $c$, were made across the entire 26-month release period.

K-means clustering was subsequently applied to the predicted trajectories of each raster cell, $\hat{Y_{c}}$, grouping them by the shape of their 26-month wMel prevalence trajectories. These cluster assignments defined the categorical outcome of the subsequent environmental regression model. Finally, the Monte Carlo Join-Count test was used to evaluate the need for a spatial smooth term by estimating spatial autocorrelation per trajectory group using False Discovery Rate (FDR)-adjusted p-values **[2]**.

The number of clusters (k=3-6) was selected using within-cluster sum of squares (WSS), average silhouette width, and bootstrap cluster stability assessed via Jaccard similarity indices. Stability was estimated by repeatedly resampling raster cell trajectory profiles, re-fitting the clustering and comparing cluster assignments to the original; mean and minimum Jaccard values were used to assess overall and worst-case reproducibility. WSS decreased monotonically with increasing k, while silhouette width peaked at k=3 and showed limiting improvement thereafter (Table S1, Fig. S1). Bootstrap stability indicated strong reproducibility for k=3-4 but substantially reduced stability for k=5-6, including poorly reproducible clusters (minimum Jaccard = 0.24 and 0.54, respectively). Although k=3 received the highest silhouette score, it merged distinct ecological trajectory types into a single broad class encompassing unsuccessful and weak intermediate replacement dynamics (Fig. S2). In contrast, k=4 separated these trajectories while maintaining high stability (mean Jaccard = 0.84; minimum Jaccard = 0.81). Higher-k solutions fragmented otherwise stable clusters without improving overall interpretability. Overall, k=4 provided the best balance between statistical fit, ecological interpretability, and cluster stability and was therefore selected for downstream analyses.

*Covariates: climate and landscape features*

A selection of 23 spatially explicit climate and landscape covariates were compiled, guided by ecological mechanisms influencing the *Ae. aegypti* and *Wolbachia* dynamics. Specifically, covariates focused on *Ae. aegypti* habitat suitability, climate sensitivity, flight barriers, and *Wolbachia* deployment barriers (Table S2).

Covariate data was obtained from publicly available sources where possible, harmonised to 500x500m spatial resolution matching the outcome variable using area-weighted areal interpolation for partially overlapping polygons and rasters, point-in-polygon summation for point features, and inverse distance-weighted interpolation for climate variables. Covariates selected for their impact on mosquito dispersal (Table S2, category: *Ae. aegypti* flight barriers) were extracted per raster cell and an additional 200m flight buffer around each raster cell, 700x700m in total. A 200m flight buffer was selected as conservative given 50–100m typical flight range of *Ae. aegypti* without the assistance of wind [3–6], yet appropriate for the municipality of Rio de Janeiro wMel release region given two studies of female *Ae. aegypti* dispersal report a maximum flight distance of 100m within densely populated Favela do Amorim [7], <10% of mosquitoes flying beyond 200m in the coastal area of Tubiacanga [7] and a mean flight distance of 288m in the residential district of Olaria [8]. Temporal resolution varied by data source, where possible data collected closest to the release period was chosen.

These covariates were then screened for zero variance and multicollinearity. Initially, the proportion of flood-prone area per raster cell (derived from Land Use / Land Class (LULC) classification) was excluded due to zero variance. Three covariates were excluded due to correlation coefficients >|0.8| [9], average literacy, building volume, and humidity. From the collinear pairs human population, the proportion of tall buildings [10–13], rainfall [14–16] and wind speed [17,18] were retained as more informative determinants of Ae. aegypti distribution. Furthermore, rainfall was interpolated from 33 weather stations across the municipality of Rio de Janeiro state relative to humidity interpolation from 7 weather stations, thus rainfall data is comparatively higher quality [19].

Covariates above the Variance Inflation Factor (VIF) high multicollinearity threshold (>10) were transformed optimising for the most informative interpretation considering research context [20–23]. Specifically, we addressed high multicollinearity by modelling non-residential buildings as a smooth function of residential buildings and retained the residuals $\varepsilon_{c}$ as the derived covariate representing the flight barrier effect of non-residential buildings. A generalised additive model (GAM) was fitted with smoothed, rather than linear, residential term as selected by AIC:

$${nonresidential}_{c}=\beta_{0}+s\left( {residential}_{c} \right)+\varepsilon_{c}$$

Environmental covariates were included as linear terms rather than smooth functions to maintain model interpretability and avoid overfitting given the relatively small sample size (n=267). Preliminary exploration indicated largely monotonic relationships with replacement outcomes, and therefore non-linear smoothing was applied only to the spatial autocorrelation term.

*Model selection: forward selection stability analysis*

To evaluate the stability of the forward AIC covariate-selection procedure, we performed a non-parametric bootstrap sensitivity analysis. Raster cells were resampled with replacement (n = 267 per bootstrap sample) and the complete forward-selection procedure was repeated independently for each bootstrap sample using the same candidate covariates, model specification, and stopping criterion (ΔAIC > 2) as the primary analysis. A total of 500 bootstrap iterations were performed.

Forward selection converged successfully for 499 of the 500 bootstrap samples (99.8%). The proportion of tall buildings (>6 m) was selected in 497 bootstrap samples (99.6%), average air temperature in 485 (97.2%), average elevation in 386 (77.4%), and average daily rainfall in 336 (67.3%). None of the remaining screened covariates were selected in any bootstrap replicate (Table S3). The order of covariate entry was also highly consistent across bootstrap samples. The proportion of tall buildings was the first covariate selected in 396 of 499 successful bootstrap samples (79.4%), followed by temperature (18.6%), while elevation (1.6%) and rainfall (0.2%) were rarely selected first. The number of retained covariates was likewise relatively stable. The full four-covariate model was recovered in 291 bootstrap samples (58.3%), three covariates were retained in 134 samples (26.9%), two covariates in 65 samples (13.0%), one covariate in 8 samples (1.6%), and no covariates met the ΔAIC selection criterion in one bootstrap sample (0.2%).

Overall, the bootstrap sensitivity analysis indicates that the principal findings are not driven by instability of the forward-selection procedure. The strongest support was consistently observed for the proportion of tall buildings and average air temperature, while elevation and rainfall showed more moderate selection stability.

**Table S1.** Cluster performance and stability across k values. WSS: within-cluster sum of squares.

| **k** | **Total WSS** | **Mean silhouette** | **Mean Jaccard** | **Minimum Jaccard** |
| --- | --- | --- | --- | --- |
| 3 | 142.67 | 0.467 | 0.867 | 0.811 |
| 4 | 110.87 | 0.392 | 0.844 | 0.805 |
| 5 | 88.67 | 0.402 | 0.668 | 0.237 |
| 6 | 73.05 | 0.408 | 0.681 | 0.540 |

**Table S2.** Model covariates. Details of ecological mechanism, spatial and temporal data resolution and source. GHSL: Global Human Settlement Layer; IBGE: Instituto Brasileiro de Geografia e Estatistica; LULC: Land Use / Land Class; OSM: OpenStreetMap.

| **Category** | **Feature** | **Ecological mechanism** | | **Data details** | **Spatial** | **Temporal** | **Source** |
| --- | --- | --- | --- | --- | --- | --- | --- |
| *Ae. aegypti* habitat suitability | Rainfall | Influences breeding site availability | [14–16] | Daily precipitation (mm) | Weather station Rio interpolation (n = 33) | 2017–2019 | ALERTA [19] |
|  | Human population |  | [13,24–26] | Total human population | Census sectors | 2022 | IBGE [27] |
|  | Household sewage accumulation |  | [28–31] | Proportion of households without sewage collection |  |  | IBGE [32] |
|  | Water bodies |  | [33,34] | Proportion LULC water bodies | Polygons (m^2^) | 2018 | DataRio [35] |
|  | Flood-prone areas |  | [36,37] | Proportion LULC flood-prone area |  |  |  |
|  | Residential buildings | Increases habitat suitability | [38,39] | Proportion LULC residential area |  |  |  |
|  | Vegetation cover |  | [24,33,40] | Proportion of LULC vegetation area |  |  |  |
|  | Breeding sites | Breeding sites | [41–44] | Breeding site count (sum of potted plants, storm drains, and water tanks) | Point coordinates | 2019–2022 | Street view imagery [45] |
|  | Household waste accumulation |  | [46–49] | Proportion households without waste collection | Census sectors | 2022 | IBGE [32] |
|  | Households without mains water connection | Domestic water storage important breeding sites | [43,50,51] | Number of households not connected to general water distribution network |  |  |  |
|  | Water tanks |  | [50–52] | Water tank density | 200x200m pixels | 2019–2022 | Satellite imagery [53] |
| *Ae. aegypti* climate sensitivity | Elevation | Impacts distribution | [54–57] | Average elevation (m above sea level) | 20x20m pixels | 2015 | IBGE [58] |
|  | Temperature | Impacts life history traits | [15,16] | Average air temperature (°C) | Weather station Rio interpolation (n = 7) | 2017–2019 | ALERTA [19] |
|  | Humidity |  | [14,15,59] | Average relative humidity (%) |  |  |  |
| *Ae. aegypti* flight barriers | Highway presence | Hinders flight and/or limits dispersal | [60–65] | Proportion of highways, vectors buffered into polygons (3.5m per lane for motorways and 3m per lane for primary and secondary highways) | Vectors (m) | 2025* | OSM [66] |
|  | Wind speed |  | [17,18] | Wind speed (kmh) | Weather station Rio interpolation (n = 7) | 2017–2019 | ALERTA [19] |
|  | Building volume |  | [38,67,68] | Total building volume | 100x100m pixels | 2018 | GHSL [69] |
|  | Non-residential building volume |  | [68,70–73] | Non-residential building volume |  |  |  |
|  | Non-residential buildings |  | [68,70–73] | Proportion LULC non-residential building classes | Polygons (m^2^) | 2018 | DataRio [35] |
|  | Tall buildings |  | [11–13,74] | Proportion buildings >6m height categories | 10x10m pixels | 2018 | GHSL [75] |
| *Wolbachia* deployment barriers | Favelas | Potential indicator for hard-to-reach communities | [76–80] | Proportion LULC favela area | Polygons (m^2^) | 2018 | DataRio [35] |
|  | Illiteracy |  | [76,81,82] | Average literacy | Census sectors | 2022 | IBGE [83] |
|  | Household income |  | [41,82,84] | Average household income | Neighbourhoods (bairros) |  | IBGE [85] |

* OpenStreetMap is continually updated, data retrieved on 31st July 2025 using the R package osmdata (v0.2.3)

**Table S3.** Bootstrap stability of forward AIC covariate selection. Selection frequencies are shown for all environmental covariates considered in model development. Covariates excluded before forward selection because of low variance or high collinearity are indicated and were not eligible for selection. Frequencies are calculated from 499 successful bootstrap replicates.

| **Covariate** | **Bootstrap selections** | **Selection frequency (%)** |
| --- | --- | --- |
| Proportion buildings >6m height categories | 497 | 99.6 |
| Average air temperature (°C) | 485 | 97.2 |
| Average elevation (m above sea level) | 386 | 77.4 |
| Average daily rainfall (mm) | 336 | 67.3 |
| Total human population | 0 | 0 |
| Proportion of households without sewage collection | 0 | 0 |
| Proportion water bodies | 0 | 0 |
| Proportion residential area | 0 | 0 |
| Proportion of vegetation area | 0 | 0 |
| Breeding site count | 0 | 0 |
| Proportion households without waste collection | 0 | 0 |
| Number of households not connected to mains water | 0 | 0 |
| Water tank density | 0 | 0 |
| Proportion of highways | 0 | 0 |
| Wind speed (kmh) | 0 | 0 |
| Non-residential building volume | 0 | 0 |
| Proportion non-residential building classes | 0 | 0 |
| Proportion favela area | 0 | 0 |
| Average household income | 0 | 0 |
| Proportion flood-prone area | Excluded prior to selection | |
| Total building volume | Excluded prior to selection | |
| Average relative humidity (%) | Excluded prior to selection | |
| Average literacy | Excluded prior to selection | |

**Fig. S1** Model selection diagnostics for k-mean clustering of *Wolbachia* replacement trajectories. A) Within-cluster sum of squares (WSS) across candidate values of k (3-6), showing monotonic decrease with increasing model complexity. B) Average silhouette width for each k, indicating optimal cluster separation at lower k but reduced gain beyond k=3. C) Bootstrap cluster stability assessed using Jaccard similarity, showing mean (solid line) and minimum (dashed line) cluster reproducibility across resamples. Horizontal dashed lines indicate interpretive thresholds for stable (≥0.75) and unstable (≤0.60) cluster solutions. Overall, k=4 provides the best balance between model fit, cluster separation, and stability.

**Fig. S2** Cluster assignment across k-values. A) Alluvial plot in which flows represent transitions of raster cells across clusters (k=3-6), colours indicate k=3 cluster allocations, and strata show cluster sizes at each k. B) Predicted proportion of wMel deployment trajectories across clusters (k=3-6).

**Fig. S3** Raster cell trajectories which were reassigned to unsuccessful. Highlighted in red are raster cell trajectories with slope < -0.005 which were assigned to moderate success and high success by k-means clustering and subsequently reassigned to unsuccessful. Black lines indicate trajectory means.

**Fig. S4** Distribution map of the proportion of tall buildings across the release area.

**Fig. S5** Observed data of selected model covariates. Panels for each selected covariate: the proportion of tall buildings (% >6m in height), average air temperature (°C), average elevation (m), and average daily rainfall (mm). Colour-coded by replacement trajectory with width of funnel indicating frequency.

**References to the Supplementary** **Materials**

[1]	Ribeiro dos Santos G, Durovni B, Saraceni V, Souza Riback TI, Pinto SB, Anders KL, et al. Estimating the effect of the wMel release programme on the incidence of dengue and chikungunya in Rio de Janeiro, Brazil: A spatiotemporal modelling study. Lancet Infect Dis 2022;22:1587–95. https://doi.org/10.1016/S1473-3099(22)00436-4.

[2]	Benjamini Y, Hochberg Y. Controlling the False Discovery Rate: A practical and powerful approach to multiple testing. J R Stat Soc Ser B Methodol 1995;57:289–300.

[3]	Moore TC, Brown HE. Estimating *Aedes aegypti* (Diptera: Culicidae) flight distance: Meta-data analysis. J Med Entomol 2022;59:1164–70. https://doi.org/10.1093/jme/tjac070.

[4]	Harrington LC, Scott TW, Lerdthusnee K, Coleman RC, Costero A, Clark GG, et al. Dispersal of the dengue vector *Aedes aegypti* within and between rural communities. Am J Trop Med Hyg 2005;72:209–20. https://doi.org/10.4269/ajtmh.2005.72.209.

[5]	Muir LE, Kay BH. *Aedes aegypti* survival and dispersal estimated by mark-release-recapture in Northern Australia. Am J Trop Med Hyg 1998;58:277–82. https://doi.org/10.4269/ajtmh.1998.58.277.

[6]	Honório NA, Silva W da C, Leite PJ, Gonçalves JM, Lounibos LP, Lourenço-de-Oliveira R. Dispersal of *Aedes aegypti* and *Aedes albopictus* (Diptera: Culicidae) in an urban endemic dengue area in the state of Rio de Janeiro, Brazil. Mem Inst Oswaldo Cruz, Rio de Janeiro 2003;98:191–8.

[7]	Maciel-De-Freitas R, Codeço CT, Lourenço-De-Oliveira R. Daily survival rates and dispersal of *Aedes aegypti* females in Rio de Janeiro, Brazil. Am J Trop Med Hyg 2007;76:659–65. https://doi.org/10.4269/ajtmh.2007.76.659.

[8]	Maciel-De-Freitas R, Lourenço-De-Oliveira R. Presumed unconstrained dispersal of *Aedes aegypti* in the city of Rio de Janeiro, Brazil. Rev Saúde Pública 2009;43:8–12.

[9]	Dormann CF, Elith J, Bacher S, Buchmann C, Carl G, Carré G, et al. Collinearity: A review of methods to deal with it and a simulation study evaluating their performance. Ecography 2013;36:27–46. https://doi.org/10.1111/j.1600-0587.2012.07348.x.

[10]	Tewari P, Guo P, Dickens B, Ma P, Bansal S, Lim JT. Associations between dengue incidence, ecological factors, and anthropogenic factors in Singapore. Viruses 2023;15:1917. https://doi.org/10.3390/v15091917.

[11]	Lau KW, Chen CD, Lee HL, Asri-Isa M, Zulfadli M, Sofian-Azirun M. Vertical distribution of *Aedes* mosquitoes in multiple storey buildings in Selangor and Kuala Lumpur, Malaysia. Trop Biomed 2013;30:36–45.

[12]	Jayathilake TA, Hasini DG, Wickramasinghe MB, de Silva BGD, Nissanka K. Oviposition and vertical dispersal of *Aedes* mosquitoes in multiple storey buildings in Colombo district, Sri Lanka. J Vector Borne Dis 2015;52:245–51.

[13]	Liao JR, Tu WC, Chiu MC, Kuo MH, Cheng HC, Chan CC, et al. Joint influence of architectural and spatiotemporal factors on the presence of *Aedes aegypti* in urban environments. Pest Manag Sci 2023;79:4367–75. https://doi.org/10.1002/ps.7634.

[14]	Lega J, Brown HE, Barrera R. *Aedes aegypti* (Diptera: Culicidae) abundance model improved with relative humidity and precipitation-driven egg hatching. J Med Entomol 2017;54:1375–84. https://doi.org/10.1093/jme/tjx077.

[15]	Tuladhar R, Singh A, Banjara MR, Gautam I, Dhimal M, Varma A, et al. Effect of meteorological factors on the seasonal prevalence of dengue vectors in upland hilly and lowland Terai regions of Nepal. Parasit Vectors 2019;12. https://doi.org/10.1186/s13071-019-3304-3.

[16]	Montgomery MJ, Harwood JF, Yougang AP, Wilson-Bahun TA, Tedjou AN, Keumeni CR, et al. The effects of urbanization, temperature, and rainfall on *Aedes aegypti* and *Aedes albopictus* mosquito abundance across a broad latitudinal gradient in Central Africa. Parasit Vectors 2025;18:135. https://doi.org/10.1186/s13071-025-06764-5.

[17]	Costa-da-Silva AL, Dye-Braumuller KC, Wagner-Coello HU, Li H, Johnson-Carson D, Gunter SM, et al. Landscape and meteorological variables associated with *Aedes aegypti* and *Aedes albopictus* mosquito infestation in two southeastern USA coastal cities. J Vector Ecol 2024;50:28–38. https://doi.org/10.52707/1081-1710-50.1-28.

[18]	Santos IC da S, Braga C, de Souza WV, de Oliveira ALS, Regis LN. The influence of meteorological variables on the oviposition dynamics of *Aedes aegypti* (Diptera: Culicidae) in four environmentally distinct areas in Northeast Brazil. Mem Inst Oswaldo Cruz 2020;115:1–10. https://doi.org/10.1590/0074-02760200046.

[19]	Centro de Operacoes Rio. Sistema Alerta Rio da Prefeitura do Rio de Janeiro [Dataset]. 2023.

[20]	Neter J, Wasserman W, Kutner MH. Applied Linear Regression Models. Homewood, IL: Inc. Search in; 1989.

[21]	Marquardt DW. Generalized inverses, ridge regression, biased linear estimation, and nonlinear estimation. Technometrics 1970;12:591–612.

[22]	O’Brien RM. A caution regarding rules of thumb for variance inflation factors. Qual Quant 2007;41:673–90. https://doi.org/10.1007/s11135-006-9018-6.

[23]	Menard S. Applied logistic regression analysis. 1st ed. SAGE publications; 1995.

[24]	Benitez EM, Sangermano F, Ludueña-Almeida FF, Almirón WR, Estallo EL. *Aedes aegypti* in the city of Córdoba: Spatial distribution and habitat suitability drivers. J Clim Change Health 2025;25:100550. https://doi.org/10.1016/j.joclim.2025.100550.

[25]	Kolimenakis A, Heinz S, Wilson ML, Winkler V, Yakob L, Michaelakis A, et al. The role of urbanisation in the spread of *Aedes* mosquitoes and the diseases they transmit – a systematic review. PLoS Negl Trop Dis 2021;15:e0009631. https://doi.org/10.1371/journal.pntd.0009631.

[26]	Rodrigues MDM, Marques GRAM, Serpa LLN, Arduino MDB, Voltolini JC, Barbosa GL, et al. Density of *Aedes aegypti* and *Aedes albopictus* and its association with number of residents and meteorological variables in the home environment of dengue endemic area, São Paulo, Brazil. Parasit Vectors 2015;8:115. https://doi.org/10.1186/s13071-015-0703-y.

[27]	Instituto Brasileiro de Geografia e Estatística (IBGE). Censo Demográfico 2022: Basico [Dataset]. 2022.

[28]	Godson A, Kumar A. Sewage-driven mosquito proliferation in Urban India: A molecular and environmental correlation study in Coimbatore. Environ Monit Assess 2025;197:1006. https://doi.org/10.1007/s10661-025-14393-9.

[29]	Durant AC, Donini A. Development of *Aedes aegypti* (Diptera: Culicidae) mosquito larvae in high ammonia sewage in septic tanks causes alterations in ammonia excretion, ammonia transporter expression, and osmoregulation. Sci Rep 2019;9. https://doi.org/10.1038/s41598-019-54413-6.

[30]	Burke RL, Barrera R, Lewis M, Kluchinsky T, Claborn D. Septic tanks as larval habitats for the mosquitoes *Aedes aegypti* and *Culex quinquefasciatus* in Playa-Playita, Puerto Rico. Med Vet Entomol 2010;24:117–23. https://doi.org/10.1111/j.1365-2915.2010.00864.x.

[31]	Mackay AJ, Amador M, Diaz A, Smith J, Barrera R. Dynamics of *Aedes aegypti* and *Culex quinquefasciatus* in septic tanks. J Am Mosq Control Assoc 2009;25:409–16.

[32]	Instituto Brasileiro de Geografia e Estatística (IBGE). Censo Demográfico 2022: Domicillo [Dataset]. 2022.

[33]	Benitez EM, Ludueña-Almeida F, Frías-Céspedes M, Almirón WR, Estallo EL. Could land cover influence *Aedes aegypti* mosquito populations? Med Vet Entomol 2020;34:138–44. https://doi.org/10.1111/mve.12422.

[34]	Nasir S, Jabeen F, Abbas S, Nasir I, Debboun M. Effect of climatic conditions and water bodies on population dynamics of the dengue vector, *Aedes aegypti* (Diptera: Culicidae). J Arthropod-Borne Dis 2017;11:50.

[35]	Prefeitura da Cidade do Rio de Janeiro. Land use. 2023.

[36]	Duchet C, Moraru GM, Segev O, Spencer M, Gershberg Hayoon A, Blaustein L. Effects of flash flooding on mosquito and community dynamics in experimental pools. Journal of Vector Ecology 2017;42:254–63. https://doi.org/10.1111/jvec.12265.

[37]	Yee SH, Yee DA, de Jesus Crespo R, Oczkowski A, Bai F, Friedman S. Linking water quality to *Aedes aegypti* and Zika in flood-prone neighborhoods. Ecohealth 2019;16:191–209. https://doi.org/10.1007/s10393-019-01406-6.

[38]	Walker KR, Joy TK, Ellers-Kirk C, Ramberg FB. Human and environmental factors affecting *Aedes aegypti* distribution in an arid urban environment. J Am Mosq Control Assoc 2011;27:135–41. https://doi.org/10.2987/10-6078.1.

[39]	Champion SR, Vitek CJ. *Aedes aegypti* and *Aedes albopictus* habitat preferences in South Texas, USA. Environ Health Insights 2014;8. https://doi.org/10.4137/EHI.S16004.

[40]	Vezzani D, Rubio A, Velázquez SM, Schweigmann N, Wiegand T. Detailed assessment of microhabitat suitability for *Aedes aegypti* (Diptera: Culicidae) in Buenos Aires, Argentina. Acta Trop 2005;95:123–31. https://doi.org/10.1016/j.actatropica.2005.03.010.

[41]	Walker KR, Williamson D, Carrière Y, Reyes-Castro PA, Haenchen S, Hayden MH, et al. Socioeconomic and human behavioral factors associated with *Aedes aegypti* (Diptera: Culicidae) immature habitat in Tucson, AZ. J Med Entomol 2018;55:955–63. https://doi.org/10.1093/jme/tjy011.

[42]	Wilke ABB, Vasquez C, Carvajal A, Medina J, Chase C, Cardenas G, et al. Proliferation of *Aedes aegypti* in urban environments mediated by the availability of key aquatic habitats. Sci Rep 2020;10. https://doi.org/10.1038/s41598-020-69759-5.

[43]	David MR, Dantas ES, Maciel-de-Freitas R, Codeço CT, Prast AE, Lourenço-de-Oliveira R. Influence of larval habitat environmental characteristics on Culicidae immature abundance and body size of adult *Aedes aegypti*. Front Ecol Evol 2021;9. https://doi.org/10.3389/fevo.2021.626757.

[44]	Paploski IAD, Rodrigues MS, Mugabe VA, Kikuti M, Tavares AS, Reis MG, et al. Storm drains as larval development and adult resting sites for *Aedes aegypti* and *Aedes albopictus* in Salvador, Brazil. Parasit Vectors 2016;9. https://doi.org/10.1186/s13071-016-1705-0.

[45]	Knoblauch S, Su Yin M, Chatrinan K, de Aragão Rocha AA, Haddawy P, Biljecki F, et al. High-resolution mapping of urban *Aedes aegypti* immature abundance through breeding site detection based on satellite and street view imagery. Sci Rep 2024;14. https://doi.org/10.1038/s41598-024-67914-w.

[46]	Mukhtar MU, Han Q, Liao C, Haq F, Arslan A, Bhatti A. Seasonal distribution and container preference ratio of the dengue fever vector (*Aedes aegypti*, Diptera: Culicidae) in Rawalpindi, Pakistan. J Med Entomol 2018;55:1011–5. https://doi.org/10.1093/jme/tjy010.

[47]	Mazine CAB, Macoris MLG, Andrighetti MTM, Yasumaro S, Silva ME, Nelson MJ, et al. Disposable containers as larval habitats for *Aedes aegypti* in a city with regular refuse collection: A study in Marilia, Silo Paulo State, Brazil. Acta Trop 1996;62:1–13.

[48]	Kenneson A, Beltrán-Ayala E, Borbor-Cordova MJ, Polhemus ME, Ryan SJ, Endy TP, et al. Social-ecological factors and preventive actions decrease the risk of dengue infection at the household-level: Results from a prospective dengue surveillance study in Machala, Ecuador. PLoS Negl Trop Dis 2017;11:e0006150. https://doi.org/10.1371/journal.pntd.0006150.

[49]	Krystosik A, Njoroge G, Odhiambo L, Forsyth JE, Mutuku F, LaBeaud AD. Solid wastes provide breeding sites, burrows, and food for biological disease vectors, and urban zoonotic reservoirs: A call to action for solutions-based research. Front Public Health 2020;7:405. https://doi.org/10.3389/fpubh.2019.00405.

[50]	Hiscox A, Kaye A, Vongphayloth K, Banks I, Piffer M, Khammanithong P, et al. Risk factors for the presence of *Aedes aegypti* and *Aedes albopictus* in domestic water-holding containers in areas impacted by the Nam Theun 2 hydroelectric project, Laos. Am J Trop Med Hyg 2013;88:1070–8. https://doi.org/10.4269/ajtmh.12-0623.

[51]	Hemme RR, Tank JL, Chadee DD, Severson DW. Environmental conditions in water storage drums and influences on *Aedes aegypti* in Trinidad, West Indies. Acta Trop 2009;112:59–66. https://doi.org/10.1016/j.actatropica.2009.06.008.

[52]	Aziz AT, Dieng H, Ahmad AH, Mahyoub JA, Turkistani AM, Mesed H, et al. Household survey of container-breeding mosquitoes and climatic factors influencing the prevalence of *Aedes aegypti* (Diptera: Culicidae) in Makkah City, Saudi Arabia. Asian Pac J Trop Biomed 2012;2:849–57. https://doi.org/10.1016/S2221-1691(12)60242-1.

[53]	Knoblauch S, Li H, Lautenbach S, Elshiaty Y, De AA, Rocha A, et al. Semi-supervised water tank detection to support vector control of emerging infectious diseases transmitted by *Aedes aegypti*. Int J Appl Earth Obs Geoinf 2023;119:103304. https://doi.org/10.11588/data/7.

[54]	Dhimal M, Gautam I, Joshi HD, O’Hara RB, Ahrens B, Kuch U. Risk factors for the presence of chikungunya and dengue vectors (*Aedes aegypti* and *Aedes albopictus*), their altitudinal distribution and climatic determinants of their abundance in Central Nepal. PLoS Negl Trop Dis 2015;9. https://doi.org/10.1371/journal.pntd.0003545.

[55]	Lozano-Fuentes S, Hayden MH, Welsh-Rodriguez C, Ochoa-Martinez C, Tapia-Santos B, Kobylinski KC, et al. The dengue virus mosquito vector *Aedes aegypti* at high elevation in México. Am J Trop Med Hyg 2012;87:902–9. https://doi.org/10.4269/ajtmh.2012.12-0244.

[56]	Ashby J, Moreno-Madriñán MJ, Yiannoutsos CT, Stanforth A. Niche modeling of dengue fever using remotely sensed environmental factors and boosted regression trees. Remote Sens (Basel) 2017;9. https://doi.org/10.3390/rs9040328.

[57]	Lippi CA, Stewart-Ibarra AM, Franklin Bajaña Loor ME, Dueñas Zambrano JE, Espinoza Lopez NA, Blackburn JK, et al. Geographic shifts in *Aedes aegypti* habitat suitability in Ecuador using larval surveillance data and ecological niche modeling: Implications of climate change for public health vector control. PLoS Negl Trop Dis 2019;13. https://doi.org/10.1371/journal.pntd.0007322.

[58]	Instituto Brasileiro de Geografia e Estatística (IBGE). Modelo Digital de Elevação (MDE) [Dataset]. 2015.

[59]	Dickens BL, Sun H, Jit M, Cook AR, Carrasco LR. Determining environmental and anthropogenic factors which explain the global distribution of *Aedes aegypti* and *Ae. albopictus*. BMJ Glob Health 2018;3. https://doi.org/10.1136/bmjgh-2018-000801.

[60]	Schmidt TL, Barton NH, Rašić G, Turley AP, Montgomery BL, Iturbe-Ormaetxe I, et al. Local introduction and heterogeneous spatial spread of dengue-suppressing *Wolbachia* through an urban population of *Aedes aegypti*. PLoS Biol 2017;15:e2001894. https://doi.org/10.1371/journal.pbio.2001894.

[61]	Schmidt TL, Filipović I, Hoffmann AA, Rašić G. Fine-scale landscape genomics helps explain the slow spatial spread of *Wolbachia* through the *Aedes aegypti* population in Cairns, Australia. Heredity (Edinb) 2018;120:386–95. https://doi.org/10.1038/s41437-017-0039-9.

[62]	Regilme MAF, Carvajal TM, Honnen AC, Amalin DM, Watanabeid K. The influence of roads on the fine-scale population genetic structure of the dengue vector *Aedes aegypti* (Linnaeus). PLoS Negl Trop Dis 2021;15:1–14. https://doi.org/10.1371/journal.pntd.0009139.

[63]	Russell RC, Webb CE, Williams CR, Ritchie SA. Mark-release-recapture study to measure dispersal of the mosquito *Aedes aegypti* in Cairns, Queensland, Australia. Med Vet Entomol 2005;19:451–7. https://doi.org/10.1111/j.1365-2915.2005.00589.x.

[64]	Hemme RR, Thomas CL, Chadee DD, Severson DW. Influence of urban landscapes on population dynamics in a short-distance migrant mosquito: Evidence for the dengue vector *Aedes aegypti*. PLoS Negl Trop Dis 2010;4:e634. https://doi.org/10.1371/journal.pntd.0000634.

[65]	Barbu CM, Hong A, Manne JM, Small DS, Quintanilla Calderón JE, Sethuraman K, et al. The effects of city streets on an urban disease vector. PLoS Comput Biol 2013;9:e1002801. https://doi.org/10.1371/journal.pcbi.1002801.

[66]	Padgham M, Lovelace R, Salmon M, Rudis B. osmdata. The Journal of Open Source Software 2017;2:305. https://doi.org/10.21105/joss.00305.

[67]	Cox J, Grillet ME, Ramos OM, Amador M, Barrera R. Habitat segregation of dengue vectors along an urban environmental gradient. Am J Trop Med Hyg 2007;76:820–6.

[68]	Carbajo AE, Curto SI, Schweigmann NJ. Spatial distribution pattern of oviposition in the mosquito *Aedes aegypti* in relation to urbanization in Buenos Aires: Southern fringe bionomics of an introduced vector. Med Vet Entomol 2006;20:209–18. https://doi.org/10.1111/j.1365-2915.2006.00625.x.

[69]	Pesaresi M, Politis P. GHS-BUILT-V R2023A - GHS built-up volume grids derived from joint assessment of Sentinel2, Landsat, and global DEM data, multitemporal (1975-2030) [Dataset]. 2023. https://doi.org/doi:10.2905/AB2F107A-03CD-47A3-85E5-139D8EC63283.

[70]	Soto-López JD, Barrios-Izás MA, Vieira Lista MC, Muro A. Role of non-residential larval habitats in *Aedes* spatiotemporal egg production 2024;14:1013. https://doi.org/10.3390/life14081013.

[71]	Dharmamuthuraja D, Rohini PD, Iswarya Lakshmi M, Isvaran K, Ghosh SK, Ishtiaq F. Determinants of *Aedes* mosquito larval ecology in a heterogeneous urban environment - a longitudinal study in Bengaluru, India. PLoS Negl Trop Dis 2023;17:e0011702. https://doi.org/10.1371/journal.pntd.0011702.

[72]	dos Reis IC, Honório NA, Codeço CT, Magalhães M de AFM, Lourenço-de-Oliveira R, Barcellos C. Relevance of differentiating between residential and non-residential premises for surveillance and control of *Aedes aegypti* in Rio de Janeiro, Brazil. Acta Trop 2010;114:37–43. https://doi.org/10.1016/j.actatropica.2010.01.001.

[73]	Morrison AC, Sihuincha M, Stancil JD, Zamora E, Astete H, Olson JG, et al. *Aedes aegypti* (Diptera: Culicidae) production from nonresidential sites in the Amazonian city of Iquitos, Peru. Ann Trop Med Parasitol 2006;100:S73–86. https://doi.org/10.1179/136485906X105534.

[74]	Fernandez SA, Sun H, Dickens BL, Ng LC, Cook AR, Lim JT. Features of the urban environment associated with *Aedes aegypti* abundance in high-rise public apartments in Singapore: An environmental case-control study. PLoS Negl Trop Dis 2023;17:e0011075. https://doi.org/10.1371/journal.pntd.0011075.

[75]	Pesaresi M, Politis P. GHS-BUILT-C R2023A - GHS Settlement Characteristics, derived from Sentinel2 composite (2018) and other GHS R2023A data [Dataset]. 2023. https://doi.org/doi:10.2905/3c60ddf6-0586-4190-854b-f6aa0edc2a30.

[76]	Ferreira AC, Chiaravalloti F, Ii N, Neto FC. Infestation of an urban area by *Aedes aegypti* and relation with socioeconomic levels. Rev Saúde Pública 2007;41.

[77]	Wermelinger ED, Salles ICM, Ferreira AP. The conflict mediation potential to control arbovirus vectors in Brazilian slums. Saúde Em Debate 2023;47:630–40. https://doi.org/10.1590/0103-1104202313819.

[78]	Charlesworth SM, Kligerman DC, Blackett M, Warwick F. The potential to address disease vectors in favelas in Brazil using sustainable drainage systems: Zika, drainage and greywater management. Int J Environ Res Public Health 2022;19:2860. https://doi.org/10.3390/ijerph19052860.

[79]	Souza RL, Nazare RJ, Argibay HD, Pellizzaro M, Anjos RO, Portilho MM, et al. Density of *Aedes aegypti* (Diptera: Culicidae) in a low-income Brazilian urban community where dengue, Zika, and chikungunya viruses co-circulate. Parasit Vectors 2023;16:159. https://doi.org/10.1186/s13071-023-05766-5.

[80]	Freitas LP, Cruz OG, Lowe R, Carvalho MS. Space–time dynamics of a triple epidemic: Dengue, chikungunya and Zika clusters in the city of Rio de Janeiro. Proceedings of the Royal Society B: Biological Sciences 2019;286:20191867. https://doi.org/10.1098/rspb.2019.1867.

[81]	Quintero J, Carrasquilla G, Suárez R, González C, Olano VA. An ecosystemic approach to evaluating ecological, socioeconomic and group dynamics affecting the prevalence of *Aedes aegypti* in two Colombian towns. Cad Saude Publica 2009;25.

[82]	Dhar-Chowdhury P, Haque CE, Lindsay R, Hossain S. Socioeconomic and ecological factors influencing *Aedes aegypti* prevalence, abundance, and distribution in Dhaka, Bangladesh. Am J Trop Med Hyg 2016;94:1223–33. https://doi.org/10.4269/ajtmh.15-0639.

[83]	Instituto Brasileiro de Geografia e Estatística (IBGE). Censo Demográfico 2022: Alfabetizacao [Dataset]. 2022.

[84]	Fustec B, Phanitchat T, Hoq MI, Aromseree S, Pientong C, Thaewnongiew K, et al. Complex relationships between aedes vectors, socio-economics and dengue transmission — lessons learned from a case-control study in Northeastern Thailand. PLoS Negl Trop Dis 2020;14:1–25. https://doi.org/10.1371/journal.pntd.0008703.

[85]	Instituto Brasileiro de Geografia e Estatística (IBGE). Censo Demográfico 2022: Rendimento do Responsável [Dataset]. Rio de Janeiro: 2022.
